# Mathematical model of mechanobiology of acute and repeated synaptic injury and systemic biomarker kinetics

**DOI:** 10.3389/fncel.2023.1007062

**Published:** 2023-02-06

**Authors:** Hamidreza Gharahi, Harsha T. Garimella, Zhijian J. Chen, Raj K. Gupta, Andrzej Przekwas

**Affiliations:** ^1^Biomedical and Data Sciences Division, CFD Research Corporation, Huntsville, AL, United States; ^2^Department of Defense Blast Injury Research Program Coordinating Office, U.S. Army Medical Research and Development Command, Fort Detrick, MD, United States

**Keywords:** traumatic brain injury, biomarkers, mechanobiology, neurobiology, synaptic damage, mathematical modeling, injury and repair, repeated blast

## Abstract

**Background:**

Blast induced Traumatic Brain Injury (bTBI) has become a signature casualty of military operations. Recently, military medics observed neurocognitive deficits in servicemen exposed to repeated low level blast (LLB) waves during military heavy weapons training. In spite of significant clinical and preclinical TBI research, current understanding of injury mechanisms and short- and long-term outcomes is limited. Mathematical models of bTBI biomechanics and mechanobiology of sensitive neuro-structures such as synapses may help in better understanding of injury mechanisms and in the development of improved diagnostics and neuroprotective strategies.

**Methods and results:**

In this work, we formulated a model of a single synaptic structure integrating the dynamics of the synaptic cell adhesion molecules (CAMs) with the deformation mechanics of the synaptic cleft. The model can resolve time scales ranging from milliseconds during the hyperacute phase of mechanical loading to minutes-hours acute/chronic phase of injury progression/repair. The model was used to simulate the synaptic injury responses caused by repeated blast loads.

**Conclusion:**

Our simulations demonstrated the importance of the number of exposures compared to the duration of recovery period between repeated loads on the synaptic injury responses. The paper recognizes current limitations of the model and identifies potential improvements.

## Graphical Abstract

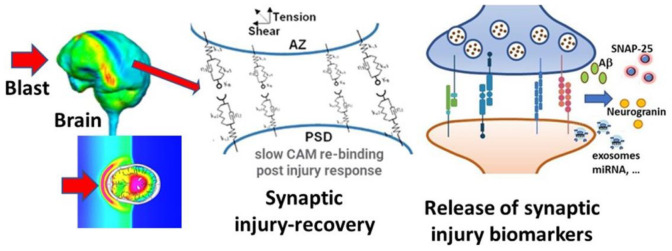

## 1. Introduction

Repeated concussions, relatively common in contact sports, have been recognized as serious medical events that can cause sustained cognitive and psychiatric changes, as well as neurodegeneration (McKee et al., 2009; Prins et al., 2013; Greco et al., 2019; Zetterberg et al., 2019; Kashyap et al., 2022). Recently, it has become evident that non-injurious sub-concussive repeated head impacts, such as frequent heading in soccer, may result in acute and chronic neurological effects (Ashton et al., 2020; Kakavas et al., 2021; McCunn et al., 2021; Sandmo et al., 2022). Blast induced traumatic brain injury (bTBI) has been referred to as a hallmark neurological signature in servicemen exposed to blast waves generated by improvised explosive devices (IED) during recent military operations (Cernak, 2017; DePalma and Hoffman, 2018;

Elder et al., 2019; Siedhoff et al., 2022). During training, military personnel may be repeatedly exposed to LLB while using breaching explosives to gain entry and firing of the heavy weapon systems such as artillery, mortars, and shoulder mounted recoilless rifles. Just as in the civilian sports, military medics observed neurocognitive deficits in servicemen exposed to repeated low level blast (LLB) waves during military heavy weapons training (Carr et al., 2015; Engel et al., 2019; Modica et al., 2021). The neurological and neurocognitive changes experienced by those who have sustained repeated LLB blast exposures may have different cellular and anatomical underpinnings compared to those with clinically-diagnosed concussion.

Following recommendations from the U.S. Congress, the U.S. Department of Defense (DoD) is taking steps to understand and mitigate any potentially harmful effects from the occupational blast exposure. The goal of that program is to register the blast pressure profiles, referred to as a “*dose*”, using wearable sensors on individual serviceman during weapon training and to collect the medical data, “*response*” at various times post-exposure. However, to date there are no clear methods to correlate the *dose* and *response* parameters that could be used for definite medical diagnostics.

**FIGURE 1:**
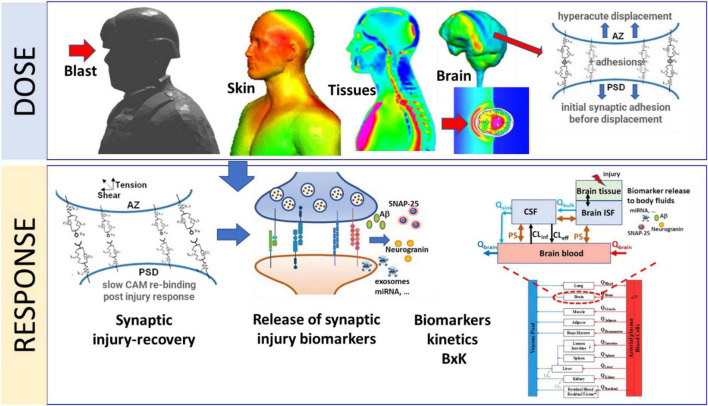
Schematic representation of major components of the *Dose-Response* modeling framework for blast induced synaptic brain injury and biomarkers kinetics. The *Dose* module computes blast induced brain macro-biomechanics and the hyper-acute micro-mechanics of synaptic adhesion molecules. The *Response* module simulates kinetics of synaptic cell adhesion molecules, synaptic damage/repair, release of synaptic biomarkers into CNS fluids and their whole-body kinetics (BxK).

Mathematical models of brain injury biomechanics (Gupta and Przekwas, 2013; Garimella and Kraft, 2017; Garimella et al., 2018), coupled to mechanobiology of injury sensitive neuroaxonal structures (Przekwas et al., 2016) and neuro signaling pathways could help in better understanding and quantitation of the dose-response challenge. Reported mathematical models of brain-scale blast-induced biomechanics can predict local brain tissue stress-strain profiles (Gupta et al., 2017). However, in spite of large volume of work on mathematical modeling of synaptic, axonal and neuronal neurotransmission, metabolism and signaling pathways very little has been reported on modeling synaptic mechanobiology (Przekwas et al., 2016; Hall et al., 2021; Keating and Cullen, 2021; Hoffe and Holahan, 2022; Procès et al., 2022). In the present paper we introduce an initial formulation of a computational model of a mechanobiological “response” of a neuronal synapse to acute and repeated sub-concussive blast loads. We focused on the mechanobiology of a synaptic cleft for its very sensitive structural morphology and its extreme metabolic and neurophysiological activity. Our goal is to develop a prototype mathematical model of the acute and repeated mechanical injury of the synaptic cleft, linked to the sub-acute/chronic synaptic biochemical responses and release of biomarkers that could be detected in body fluids. This model will be introduced as a part of the computational framework, CoBi-Neuro, linking the *dose* represented by blast induced brain scale biomechanics and the micro-mechanics of injury sensitive neuro-structures and the *response* simulated by mechanobiology of these structures, release of injury biomarkers, and their bio-distribution in human body fluids, Figure 1.

Due to the anatomical and neurological complexity of the human brain and wide range of spatial and temporal scales such computational model inherently involves various approximations and assumptions (Gupta et al., 2017). Development and validation of such a model requires experimental data for the *dose* and the *response* model components. Reliable experimental data of blast wave loads on the human physical surrogate body/head can be collected and used to validate the *dose* model. Moreover, the blast *dose* on the US servicemen involved in weapon training can be computed using the wearable pressure sensor and weapon blast signature data (Przekwas et al., 2021). Acquisition of direct and objective data for the development and validation of the human neuro-*response* model is not feasible. Experimental data from animal models and *in-vitro* devices such as brain-on-chip, could provide some of the mechanobiological data but the translation to humans is debatable. However, medical imaging and body fluid biomarkers data can provide an indirect data for the development and validation of the neuro-*response* model. In this work we use brain injury biomarkers collected from the body fluids at various time points post-blast exposure (Gupta et al., 2017) to develop a prototype neuro-*response* model. Figure 1 schematically identifies major components of the CoBi-Neuro repeated brain injury dose-response framework.

## 2. Materials and methods

### 2.1. Mechanobiology of synaptic injury: Model description

We first present a novel mathematical framework to study temporally multiscale synaptic damage mechanisms. In this work, we use the term synapse as a short-hand for the chemical synapses in the brain. Synapses are comprised of three main components. First, a pre-synaptic membrane which contains a high concentration of mitochondria and neurotransmitter vesicles. Second, synaptic cleft is the gap between two nerve cells across which neurotransmitters diffuse after release from vesicles. Third, a post-synaptic membrane that contains receptor sites to which neurotransmitters bind leading to permeability change that produces the post-synaptic potential.

Motivated by models of immunological synapse assembly (Qi et al., 2001), we postulate that the configuration of the synapse is governed by the mechanical inter-membrane separation distance that drives the system to the minimum free energy functional. We assume that the pre- and post-synaptic membranes form two parallel rigid planes, where the synaptic cleft size is moderated *via* cell adhesion molecules (CAMs) complexes. CAMs populate the surface of the pre- and post-synapse, bind to their counterparts on the opposite membrane, and form a spring-damper-type network that structurally supports the synapse structure (Przekwas et al., 2016). In fact, CAM complex (re)formation, modulated by binding and dissociation of individual CAMs, plays a pivotal role in the response of synapse to a forced membrane separation (e.g., in blast-induced deformations). The ability of the CAMs to form complexes following a separation depends on their distance and the force acting on their inter-molecular bond. In addition to CAM-CAM interactions, connection of a CAM to the pre- or post-synaptic membrane may also play a role in synaptic damage and recovery. For instance, a fast separation of the synaptic membranes may lead to detachment of some CAMs from their cytoplasmic anchor, effectively inhibiting their rebinding.

More than twenty different families of synaptic CAMs have been identified with diverse adhesive behaviors, calcium sensitivities, and functions (Bukalo and Dityatev, 2012). However, only three representative families of CAM complexes are considered in this work to reduce the model and parameter complexities: (1) Neurexin:Neuroligin, (2) Synaptic CAMs (SynCAMs), and (3) N-Cadherins (NCADs). Neurexins (pre-synaptic) and neuroligins (post-synaptic) are CAMs that trans-synaptically interact to provide connectivity between membranes and ensure proper synaptic functions. In addition to providing mechanical support, neurexins (NX) and neuroligins (NL) play crucial roles in neurotransmission and differentiation of synapses, as well as maintaining NMDA and AMPA receptor function (Heine et al., 2008). Pathologically, NX-NL complexes (XL) are involved in amyloid β (Aβ) synaptotoxicity in Alzheimer’s disease (Brito-Moreira et al., 2017). SynCAM1-4 are immunoglobin proteins that constitute a family of CAMs central in synaptogenesis (Fowler et al., 2017). In addition, although SynCAM1-4 can form homophilic bonds, they preferentially assemble into heterophilic complexes (Fogel et al., 2007). Lastly, NCADs are a subgroup of the super-family of Ca^2+^-dependent CAMs that homophilically bind together to form the NCAD:NCAD complex and contribute to formation stability of the synapses (Arikkath and Reichardt, 2008). The cytoplasmic end of cadherins form a strong catch bond with catenins where force strengthens the bound state (Buckley et al., 2014).

### 2.2. Mechanobiology of synaptic injury: Model formulation

In this section, we present the mathematical model of the dynamics of synaptic injury and recovery and binding/unbinding of CAMs constrained in pre- and post-synaptic membranes. The model is described by a set of ordinary differential equations (ODEs). In all the formulations we assume that thermal noise is negligible compared to other terms. In addition, we assume a uniform distribution of synaptic CAMs on the membrane surfaces and neglect their diffusion.

Our model has been adapted from an immunological synapse model (Qi et al., 2001) which accounts for intra-membrane diffusion of both receptors and complexes. Such model becomes multidimensional (at least 2D axisymmetric). It was feasible to calibrate that model, lasting several minutes, using *in vitro* imaging data. We decided to simplify the immune synapse model for two reasons: (1) in the neuronal synapse there is a myriad of other transmembrane proteins that would complicate the diffusion model, and (2) at present there is lack of data for calibration of the diffusive transport of adhesion molecules and their complexes. We have also neglected interstitial fluid flow in the synaptic cleft during sudden mechanical extension and slow recoil.

*Neurexin-Neuroligin Reactions:* The kinetics of NX, NL, and their complex XL are expressed using the reaction equations


(1) $$\frac{d\,C_{N\,X}}{d\,t}=-k_{o\,n,X\,L}\cdot C_{N\,X}\cdot C_{N\,L}+k_{o\,f\,f,X\,L}\,\left(f_{X\,L}\right)\cdot C_{X\,L}$$

$$-k_{r\,u\,p}^{N\,X}\,C_{N\,X}+k_{s\,y\,n}^{N\,X},$$

(2) $$\frac{d\,C_{N\,L}}{d\,t}=-k_{o\,n,X\,L}\cdot C_{N\,X}\cdot C_{N\,L}+k_{o\,f\,f,X\,L}\,\left(f_{X\,L}\right)\cdot C_{X\,L}$$

$$-k_{r\,u\,p}^{N\,L}\,C_{N\,L}+k_{s\,y\,n}^{N\,L},\text{and}$$

(3) $$\frac{d\,C_{X\,L}}{d\,t}=+k_{o\,n,X\,L}\cdot C_{N\,X}\cdot C_{N\,L}-k_{o\,f\,f,X\,L}\,\left(f_{X\,L}\right)$$

$$\cdot C_{X\,L}-\underset{d\,o\,w\,n\,r\,e\,g\,u\,l\,a\,t\,i\,o\,n}{\underbrace{\left(1-P_{X\,L}\right)\,k_{b\,r\,e\,a\,k,X\,L}\,C_{X\,L}}},$$

where *C*_*NX*_, *C*_*NL*_, and *C*_*XL*_ are surface concentrations of NX, NL, and XL, respectively. The unbinding rate *k_off_*_,*XL*_ varies with force acting on the intermolecular bond (Bell, 1978)


(4) $$k_{o\,f\,f,X\,L}=k_{o\,f\,f,X\,L}^{0}\cdot e^{\frac{f_{X\,L}}{f_{0,X\,L}}},$$

where $k_{o\,f\,f,X\,L}^{0}$ is the baseline unbinding rate, *f*_*XL*_ is the total force generated in XLs during synapse separation. Total force is normalized by a reference force *f*_*0,XL*_which we define as


(5) $$f_{0,X\,L}=N_{X\,L}\,\frac{K_{B}\,T}{x_{X\,L}}$$

where *N*_*XL*_the total number bound XLs, *K*_*B*_ and *T* are Boltzmann’s constant and temperature, respectively, and *x*_*XL*_ is the distance between potential energies of the intact and broken states (Ahmadzadeh et al., 2015). The binding rate is assumed to follow a Gaussian distribution centered around optimal cleft distance (*z_0_*)


(6) $$k_{o\,n,X\,L}=k_{o\,n,X\,L}^{0}\cdot e^{-\frac{{\left(z-z_{0}\right)}^{2}}{2\,\sigma^{2}}},$$

with baseline $k_{o\,n,X\,L}^{0}$ and distribution width σ. Furthermore, a portion of XL complexes may detach from their cytoplasmic connections during the insult. There are two potential parametrizations for this CAM-membrane detachment: pulling rate dependent and rupture force dependent. In this work, we use the pulling rate as the determining factor for the CAM detachment from membrane assuming that the detachment only occurs during the insult (separation of the cleft). We must note that rate dependent surface detachment are widely studied in adhesive viscoelastic material research (Violano et al., 2021). This detachment is represented by the “down-regulation” term in Eq. (3), where *P*_*XL*_is defined as


(7) $$P_{X\,L}=e^{-\frac{s\,\left(t\right)}{v_{b\,r\,e\,a\,k,X\,L}}}.$$

In this formulation, pulling rate, *s*(*t*), is normalized by a factor *v*_*break,XL*_. We assume that this down-regulation only affects the CAM complexes since the individual unbound CAMs can move with their base membrane during the insult. During a slow pulling process, the down-regulation term will vanish (*P*_*XL*_ ≈ 1), while during a fast pulling, this term becomes dominant (*P*_*XL*_ ≈ 0). Moreover, we assume that the NX and NL are produced ($k_{s\,y\,n}^{N\,X}$ and $k_{s\,y\,n}^{N\,L})$ and degraded ($k_{r\,u\,p}^{N\,X}$ and $k_{r\,u\,p}^{N\,L}$) at a constant rate. Therefore, the damage induced by the fast pulling can be remedied *via* natural turnover of NX and NL over time. However, we will refer to this damage as irreversible with regards to the timescale of our analysis here. We must note that the stoichiometry of NL is 2:2 (Comoletti et al., 2008), however, their dimer concentrations are considered in this work.

*SynCAM Compartment (the complex is SynC12):* SynCAM1 preferentially binds SynCAM2 to form a complex (Fogel et al., 2010), hence we use SynC12 for their complex. However, for simplicity we assume that SynCAM1 and SynCAM2 have the same properties and concentrations. Therefore, the kinetics of SynCAM and associated complex SynC12 can be written as


(8) $$\frac{d\,C_{S\,y\,n\,C}}{d\,t}=-k_{o\,n,S\,y\,n\,C}\cdot C_{S\,y\,n\,C}^{2}+k_{o\,f\,f,S\,y\,n\,C}\,\left(f_{S\,y\,n\,C\,12}\right)\cdot C_{S\,y\,n\,C\,12}$$

$$\;-k_{r\,u\,p}^{S\,y\,n\,C}\,C_{S\,y\,n\,C}+k_{s\,y\,n}^{S\,y\,n\,C},$$

(9) $$\frac{d\,C_{S\,y\,n\,C\,12}}{d\,t}=+k_{o\,n,S\,y\,n\,C}\cdot C_{S\,y\,n\,C}^{2}-k_{o\,f\,f,S\,y\,n\,C}\,\left(f_{S\,y\,n\,C\,12}\right)\cdot C_{S\,y\,n\,C\,12}$$

$$\;-\left(1-P_{S\,y\,n\,C\,12}\right)\,k_{b\,r\,e\,a\,k,S\,y\,n\,C\,12}\,C_{S\,y\,n\,C\,12}.$$

Similar to Neurexin-Neuroligin compartment, *C*_*SynC*_ and *C_SynC_*_12_ are surface concentrations of SynCAM and SynC12, respectively. Moreover, the corresponding unbinding rate, binding rate, and down-regulation factor are defined as


(10) $$k_{o\,f\,f,S\,y\,n\,C}=k_{o\,f\,f,S\,y\,n\,C}^{0}\cdot e^{\frac{f_{S\,y\,n\,C}}{f_{0,S\,y\,n\,C\,12}}},$$

(11) $$\,k_{o\,n,S\,y\,n\,C}=k_{o\,n,S\,y\,n\,C}^{0}\cdot e^{-\frac{{\left(z-z_{0}\right)}^{2}}{2\,\sigma^{2}}},a\,n\,d$$

(12) $$P_{S\,y\,n\,C\,12}=e^{-\frac{s\,\left(t\right)}{v_{b\,r\,e\,a\,k,S\,y\,n\,C}}},$$

where *f*_*SynC*_ is the total force in SynC12 CAMs, $k_{o\,f\,f,S\,y\,n\,C}^{0}$ and $k_{o\,n,S\,y\,n\,C}^{0}$ are respectively the baseline unbinding and binding rates, and *v*_*break,SynC*_ is a pulling-rate normalization factor. Furthermore, a reference force is defined as


(13) $$f_{0,S\,y\,n\,C\,12}=N_{S\,y\,n\,C\,12}\,\frac{K_{B}\,T}{x_{S\,y\,n\,C\,12}}$$

where *N*_*SynC12*_ and *x*_*SynC12*_ are similar to their counterparts in Eq. (5).

*NCAD Compartment:* NCADs homophilically bind together to form the NCAD:NCAD complex. The cytoplasmic end of cadherins form a strong catch bond with catenins where force strengthens the bound state (Buckley et al., 2014). Thus, we ignore the detachment (down-regulation) term for NCAD and NCAD:NCAD kinetics


(14) $$\frac{d\,C_{N\,C\,A\,D}}{d\,t}=-k_{o\,n,N\,C\,A\,D}\cdot C_{N\,C\,A\,D}^{2}+k_{o\,f\,f,N\,C\,A\,D}\left(f_{N\,C\,A\,D:N\,C\,A\,D}\right)\cdot$$

$$C_{N\,C\,A\,D:N\,C\,A\,D}-k_{r\,u\,p}^{N\,C\,A\,D}\,C_{N\,C\,A\,D}+k_{s\,y\,n}^{N\,C\,A\,D},$$

(15) $$\frac{d\,C_{N\,C\,A\,D:N\,C\,A\,D}}{d\,t}=+k_{o\,n,N\,C\,A\,D}\cdot C_{N\,C\,A\,D}^{2}$$

$$\;\;-k_{o\,f\,f,N\,C\,A\,D}\,\left(f_{N\,C\,A\,D:N\,C\,A\,D}\right)\cdot C_{N\,C\,A\,D:N\,C\,A\,D}.$$

The concentration of unbound NCADs on the pre- and post-synaptic membranes are assumed to be equal and represented by *C*_*NCAD*_. Similar to the other CAMs, the binding and unbinding rates are


(16) $$k_{o\,f\,f,N\,C\,A\,D}=k_{o\,f\,f,N\,C\,A\,D}^{0}\cdot e^{\frac{f_{N\,C\,A\,D:N\,C\,A\,D}}{f_{0,N\,C\,A\,D}}},$$

$$k_{o\,n,N\,C\,A\,D}=k_{o\,n,N\,C\,A\,D}^{0}\cdot e^{-\frac{{\left(z-z_{0}\right)}^{2}}{2\,\sigma^{2}}}$$

where *f*_*NCAD:NCAD*_ is the total force in NCAD:NCAD complexes, $k_{o\,f\,f,N\,C\,A\,D}^{0}$ and $k_{o\,n,N\,C\,A\,D}^{0}$ are respectively the baseline unbinding and binding rates.

*Mechanics of Synaptic Adhesion:* Following (Qi et al., 2001), the free energy functional corresponding to CAM complex deformations can be written as penalties associated with bond deformation


(17) $$F=\sum_{i}\frac{\lambda_{i}}{2}\,C_{i}\,\left(t\right)\,{\left(z\,\left(t\right)-z_{0,i}\right)}^{2},i\in \left\{X\,L,S\,y\,n\,C\,12,N\,C\,A\,D:N\,C\,A\,D\right\}$$

where λ_*i*_ is the curvature of binding energy well for *i*th complex, and *z*(*t*) and *z*_*0,i*_ are the synaptic cleft and optimal complex heights (all equal to *z_0_* here), respectively. Assuming a constant λ_*i*_, total force as a function of CAM complex deformation and concentration is


(18) $$f=\sum_{i}f_{i},=\sum_{i}\lambda_{i}C_{i}\left(t\right)\left(z\left(t\right)-z_{0,i}\right).$$

**FIGURE 2:**
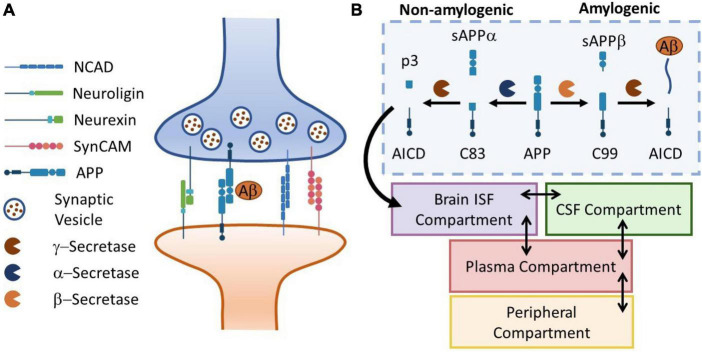
Panel **(A)** the schematic representation of a neuronal synapse with four representative families of CAMs. Aß release and kinetics is used to drive the biomarker kinetics model. Panel **(B)** the APP cleavage pathways in brain ISF diverge in to amylogenic and non-amylogenic braches, depending on the activity of sBACE (ß-secretase) and ADAM10 (α-secretase), respectively. Here, a simple four-compartment model is used to simulate transport of Aß peptides in the body. Temporal kinetics of synaptic injury biomarkers can be monitored in body fluids (plasma, CSF, saliva, …).

Finally, the time evolution of synaptic cleft distance *z*(*t*) is described by a time-dependent equation, as a functional derivative of the free energy, input displacement and *s*(*t*),


(19) $$\frac{d\,z}{d\,t}=-M\,\frac{\delta \,F}{\delta \,z}+s\,\left(t\right),$$

where *M* is a phenomenological constant for membrane response to free energy. The synaptic response to an insult is modeled as a two-step process: (1) a fast injury marked by a forced separation of pre- and post-synaptic membranes induced by tension and shear [*s*(*t*) = 0], and (2) synaptic release [*s*(*t*) = 0] characterized a relatively slow recovery of the synapse where the synaptic CAMS form bounds and pull the membranes together. The configuration of the synaptic cleft and associated CAM complexes are shown in Figure 2A. The full implementation of ODEs are provided in Supplementary material A.

### 2.3. Biomarker kinetics model description

In this section, we demonstrate the utility of our model in simulating the release and distribution of injury biomarkers in human body fluids. Several families of synaptic CAMs share common effectors with neurodegenerative diseases such as Alzheimer’s disease (AD). For instance, Presenilin-1 (PS1), the catalytic subunit of γ-secretase (a main component of Amyloid ß production), regulates the processing of neurexins (Saura et al., 2011). Moreover, animal studies have shown elevated amyloid precursor protein (APP) levels (Edwards et al., 2017) following exposure to repeated mild TBI. Interestingly, increasing evidence suggest that APP itself is a CAM (Sosa et al., 2017). Motivated by such observations, we incorporate our mechanobiology model in a biomarker kinetics framework. In this work, we use Aß_42_ production as an illustrative example. To describe the release and distribution of Aß_42_ in the body, we use a 4-compartment model adapted from Madrasi et al. (2021), where Aß_42_ is produced in brain interstitial fluid (ISF) and peripheral compartments. Moreover, we neglect the oligomerization process in the brain ISF. Figure 2B shows the schematic of APP processing pathways, Aß_42_ generation, release, transport and bio-distribution in the body (section “2.3. Biomarker kinetics model description”).

To link the mechanobiology model to the biomarker release model, we define a phenomenological function quantifying the long-term synaptic dysfunction based on the perturbation of CAM complex concentrations from their homeostatic baseline


(20) $$S\,D\,\left(t\right)=w_{1}\,\frac{C_{X\,L}\,\left(t\right)-C_{X\,L}^{0}}{C_{X\,L}^{0}}+w_{2}\,\frac{C_{S\,y\,n\,C\,12}\,\left(t\right)-C_{S\,y\,n\,C\,12}^{0}}{C_{S\,y\,n\,C\,12}^{0}}$$

$$\;\;+w_{3}\,\frac{C_{N\,C\,A\,D:N\,C\,A\,D}\,\left(t\right)-C_{N\,C\,A\,D:N\,C\,A\,D}^{0}}{C_{N\,C\,A\,D:N\,C\,A\,D}^{0}},$$

where *w_1_*, *w_2_*, and *w_3_* are the weights associated with each CAM complex and $C_{i}^{0}$ are the initial (equilibrium) concentrations. We assume that the synaptic dysfunction linearly elevates the APP synthesis rate in the brain ISF, i.e.,


(21) $$K_{B\,I\,S\,F,s\,y\,n\,t\,h}=K_{B\,I\,S\,F,s\,y\,n\,t\,h}^{0}+K_{S\,D}\,S\,D\,\left(t\right),$$

where $K_{B\,I\,S\,F,s\,y\,n\,t\,h}^{0}$ is the baseline rate and *K_SD_* is a constant. The full description of ODEs and parameters for Aß_42_ release and production are provided in Supplementary material B.

### 2.4. Parameter selection and global sensitivity analysis

The parameters of the mechanobiology model are adopted or inferred from the available data literature. Table 1 summarizes the parameters, their nominal value, their description, the reference used for parameters, and the experiment used in the reference.

**TABLE 1:** List of parameters in the mechanobiology model.

Parameter	Nominal value		Unit	Description	Note	Relevant references
*z_0_*	0.02		μm	Optimal cleft size		Gabbiani and Cox, 2010
$C_{i}^{0}$	NX/NL	140	molec/μm^2^	Equilibrium concentrations of CAMs	Quantum dot experiments Solution-based experiments	Saint-Michel et al., 2009 (NL:NX) Perret et al., 2002 (NCAD) Fogel et al., 2010 (SynCAM)
	SynCAM	20				
	NCAD	200				
*k*_*rup*_ (All CAMs)	NX/NL	3.09 × 10^–6^	1/s	Degradation, synthesis rate of proteins	Inferred from steady state concentrations and half lives	Cohen et al., 2013
	SynCAM	3.09 × 10^–6^				
	NCAD	3.50 × 10^–6^				
$k_{o\,f\,f}^{0}$	NX/NL	0.015	1/s	Equilibrium unbinding rate	Quantum dot experiments Solution-based experiments	Saint-Michel et al., 2009 (NL:NX) Perret et al., 2002 (NCAD) Fogel et al., 2010 (SynCAM)
	SynCAM	0.015				
	NCAD	0.45				
$k_{D}^{0}$	NX/NL	50	μm^2^/molec	Equilibrium dissociation constant, used to compute binding rates		
	SynCAM	200				
	NCAD	0.9				
*f_0_*	NX/NL	4,000	pN	Force associated with persistence length (*x_0_*) summed for total number of CAMs	Atomic force microscope cantilevers Flow-chamber analysis	Perret et al., 2002; Mikulska et al., 2014
	SynCAM	500				
	NCAD	4,000				
*k*_*break*_	XL and SynC12s	10	1/s	Parameters controlling detachment of CAMs from cells due to strain	Assumed	
*v*_*break*_	1		μm/s		Assumed	
σ	0.013		μm	Width distribution of binding constant	Assumed	Qi et al., 2001
λ_*CAMs*_	1250		pN.molec/ μm	Stiffness of CAMs	Assumed	
*M*	10^–8^		μm^2^/s	Phenomenological constant for synapse response to free energy changes	Assumed	Qi et al., 2001

We use a Sobol sensitivity analysis technique to measure the contribution of each parameter and its interactions with other parameters to the output variance. Excluding *z_0_* and equilibrium concentrations ($C_{i}^{0})$from the sensitivity analysis, a total of 18 parameters were used in the sensitivity analysis. To incorporate the inherent large uncertainty in the biological model parameters, each parameter is varied from 0.01 to 100 times the nominal value with a log-uniform distribution. The area-under-curve (AUC) of synaptic dysfunction following an acute insult, with *w*_1_ = *w*_2_ = *w*_3_ = 1 is used for this analysis. To implement the Sobol method, a Saltelli’s (Saltelli et al., 2010) sampling scheme was used with a total of 38,912 model evaluations. The sample generation and calculation of total Sobol index was performed *via* open-source Python’s SALib (Herman and Usher, 2017).

## 3. Results

### 3.1. Acute response to LLB exposure and global sensitivity analysis

An acute exposure to LLB is simulated *via* a forced separation of pre- and post-synaptic membranes with a pulling rate of 0.01 μm/ms, cleft size 0.02 μm and duration of 0.5 ms, i.e., equivalent to a strain rate of 500 1/s ($s=\frac{0.01\,\mu \,m/\mathrm{ms}}{0.02\,\mu \,\text{m}}$ 500 1/s), which of the same order of magnitude as computed in finite element models of blast-induced brain injury (Panzer et al., 2012). Once the injury duration is over, the synapse is released and allowed to slowly recover *via* rebinding of CAMs.

**FIGURE 3:**
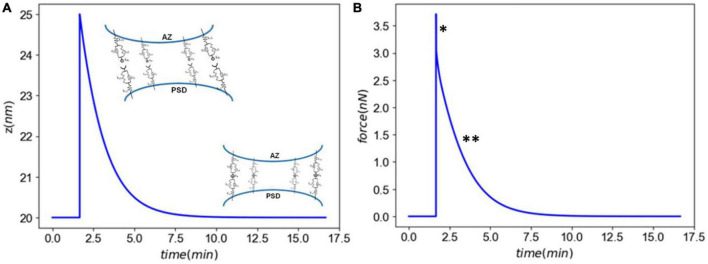
Panel **(A)** the synaptic cleft distance marked by a fast injury (∼ms) and slow recovery (∼minutes). Panel **(B)** the total force generated in CAMs marked by a a viscoelastic-type response. The astrics (*) and (^**^) delineate different modes of force generated by CAMs.

Figure 3 shows the synaptic response to an acute injury, characterized by a fast separation of membranes followed by a slow recovery. Synaptic cleft recovers its original size after a few minutes. In addition, the model shows a sharp increase in the total force generated in CAMs followed by a slow decrease, similar to a viscoelastic response. In the physiological conditions the adhesion molecules linking the pre- and post-synaptic terminals will be under an initial tension force. Our model, derived from the immune synapse model, assumes that the force is relative to the initial state.

**FIGURE 4:**
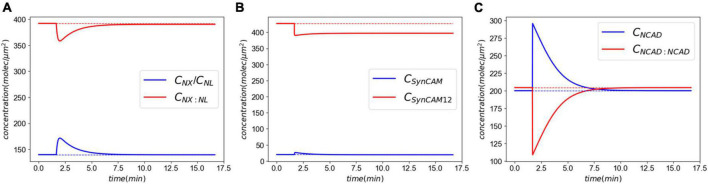
Molecular response to an acute insult. Panel **(A)** shows the XL and its constitutive CAMs response. The initial concentrations are almost fully recovered minutes after the insult. Panel **(B)** shows the SynCAM response. A clear loss of SynCAM12 complexes are predicted as a result of the injury. This loss will be remedied days-months *via* natural degradation and synthesis of SynCAMs. Conversely, the loss of NCADs minutes after the insult panel **(C)** due to lack of the pulling rate dependent down-regulation.

Figure 4 shows the CAM responses to the insult. The XL complexes almost return back to their normal levels minutes after injury (Figure 4A). However, a closer examination shows that the insult causes a slight, yet sustained, loss of CAM complexes. This loss is more pronounced in SynCAM12 complexes. In both XL and SynC12 cases, the loss will be remedied by the replenishment of lost proteins *via* the natural synthesis and degradation process over days-months after the insult (Supplementary material C). As discussed in the “2. Materials and methods” section, the NCADs create a strong catch bond with catenins at their cytoplasmic end. Therefore, they will not detach from the membranes and are able to fully rebind within minutes after the injury.

Importantly, the mechanobiology model of synaptic damage and recovery captures responses multiple timescales: (1) the <1 ms of injury, (2) minutes of partial recovery mediated by rebinding of dissociated proteins, and (3) days-months of full recovery facilitated by the natural synthesis of CAMs.

**FIGURE 5:**
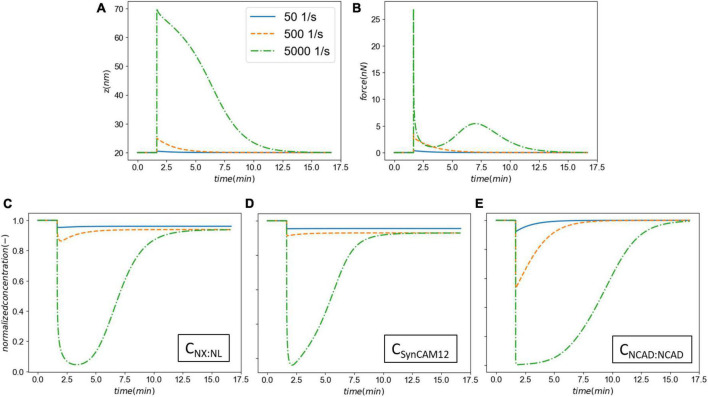
The effect the strain rate on the synaptic cell adhesion molecules (CAM) mechanics and concentrations. Panel **(A)** shows the cleft size before, during and after the insult. Panel **(B)** shows the total force applied on the intermolecular bonds. Panels **(C–E)** show the normalized CAM complex concentrations for NX:NL, SynCAM12, and NCAD:NCAD, respectively.

To further evaluate the model behavior, we varied the synaptic membrane pulling rate (*s*) across three orders of magnitude leading to strain rates 50–500–5,000 1/s while keeping the insult duration constant. Figure 5 shows the results of this parametric study. A strain rate of 50 1/s leads to a relatively small 0.5 nm cleft separation. However, even with a small separation, our model predicts a degree of CAM loss as seen on panels Figures 5C–E. As the strain rate is increased 10- and 100-fold, the synaptic cleft separation elevates to 5 and 50 nm, respectively. This separation leads to a significant drop in the concentrations of CAM complexes and delays their recovery (*via* rebinding) time. The long-term loss of SynCAM12 complexes is 4.4, 7.0, and 7.1% for smallest, medium, and largest strain rates, respectively, suggesting that our model and parameters produce a saturated CAM loss response as a function of strain rates. Interestingly, the total force complexes display a multipeak behavior when strain rate is increased to 5,000 1/s (Figure 5B). The first peak, consistent with smaller strain rates, pertains to the fast CAM complex stretch during the insult. During the recovery phase of the synapse, the CAMs rebind and pull the membranes together, where we have assumed a Gaussian function for this cleft distance-dependent rebinding with a 13 nm spread (Eq. 6). The lower-level strain rates (50 and 500 1/s) do not move the synaptic cleft outside of the Gaussian spread, whereas the strain rate of 5,000 1/s causes the rebinding to reduce to almost zero. At zero rebinding, the only driver for recovery is the energy stored in the remaining CAM complexes. However, once the synapse recovers the rebinding accelerates following the Gaussian curve while the CAM complexes are still stretched. This leads to a secondary peak in the force shown on Figure 5B. We must note that a 50 nm cleft separation will likely lead to an irrecoverable damage to synapses which is out of the scope of the current model. Nevertheless, this parametric study demonstrates an extreme model behavior which may be seen in mild-strong strain rates (500–5,000 1/s).

**FIGURE 6:**
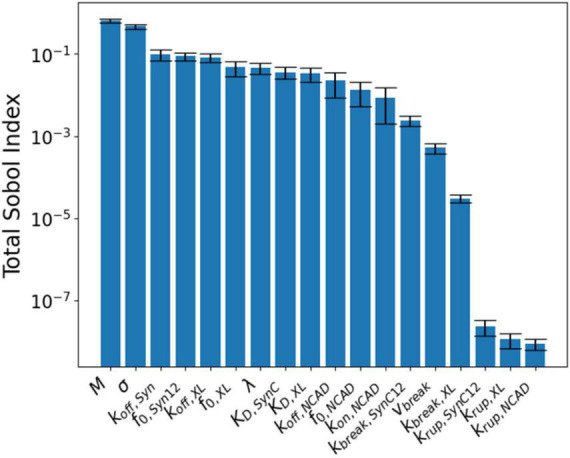
Global sensitivity analysis results ranked according to their total-order Sobol index (*S*_*T*_).

Figure 6 shows the resulting parameter sensitivities ranked based on the total-order Sobol index. The most sensitive parameters are *M* and σ. Parameter *M* corresponds to the overall response of synapse and to a large degree controls the synaptic recovery time course. Similarly, parameter σ is the spread if the Gaussian used for the binding rates, shared across all CAMs. These parameters are followed by the parameters controlling the unbinding of SynCAM and XL complexes. Due to lack of insult-induced long-term (∼days to months) loss of NCADs, its associated parameters rank lower compared to their XL and SynCAM counterparts. The least sensitive parameters based on this analysis are parameters defining the natural turnover of CAMs.

### 3.2. Synaptic damage due to exposure to repeated LLBs

**FIGURE 7:**
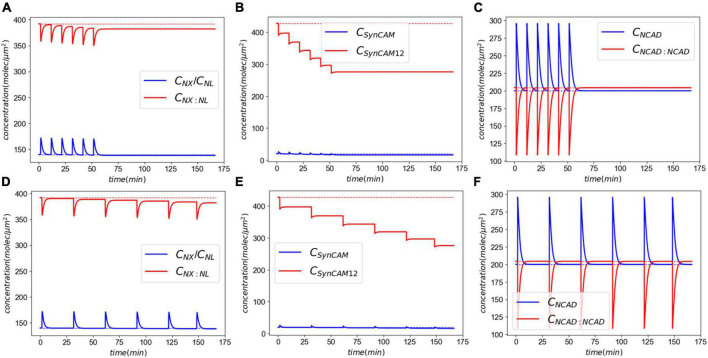
Molecular response to exposure to repeated LLBs. Panels **(A–C)** show the response to six insults every 10 min. Panels **(D–F)** show the response to six insults every 30 min.

In this section, we extend the acute response model to simulate exposure to repeated LLBs. We consider six identical insults (pulling rate of 0.01 μm/ms and duration of 0.5 ms) in two exposure scenarios, Case 1: insults every 10 min, and Case 2: insults every 30 min. Figure 7 shows the results of exposure to repeated blasts. The loss of XL and SynCAM complexes has markedly increased due to repeated injuries with a 2.6 and 35% loss in XLs and SynCAMs, respectively, 10 min after the last insult for both cases. These losses remain almost constant in the timescale of hours after the insults. Similar to the acute response, slow recovery of detached CAMs is due to their long half-lives and slow synthesis rates (Cohen et al., 2013). In contrast, the loss of NCADs is 0.25% after 10 min which drops to 0.08% after 20 min. The NCAD response is largely modulated by rebinding of homophilic CAMs.

### 3.3. Biomarker release and distribution following exposure to LLBs

**FIGURE 8:**
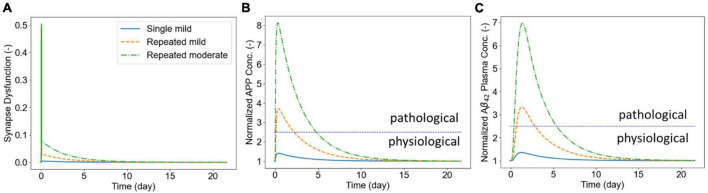
Panel **(A)** shows the synaptic dysfunction. Panel **(B)** shows the elevation in APP following the exposure to LLBs. Panel **(C)** shows the plasma concentrations of Aß_42_ following exposure to repeated insults.

Next, we show illustrative results of the biomarker release kinetics following repeated blast exposure. Our APP processing model simulates generation of both the amylogenic (sAPPβ, Aβ40, Aβ42) and non-amylogenic (sAPPα, p3) peptides that may be detected in body fluids. Here we only report results for generation of APP and Aß_42_ peptides and their kinetics in plasma for which human data have been recently reported (Boutté et al., 2021, 2022). We set *w*_1_ = 1, *w*_2_ = 0.1, and *w*_3_ = 0 in the synaptic dysfunction model [Eq. (20)]. In addition, we assumed that $K_{S\,D}=10=K_{B\,I\,S\,F,s\,y\,n\,t\,h}^{0}$. The rest of the parameters for this model were taken from Madrasi et al. (2021). The biomarker release and kinetics are simulated for 21 days following the exposure to single or repetitive blasts. Three scenarios were simulated in this section: (1) single mild insult (pulling rate of 0.01 μm/ms and duration of 0.5 ms), (2) six repeated mild insults, and (3) six repeated moderate insults (pulling rate of 0.04 μm/ms and duration of 1.0 ms). Figure 8A shows the synaptic dysfunction for the three cases. The spikes in this figure correspond to dynamics of short-term response of CAMs (shown in Figure 7) that are followed by a slower decline in synaptic dysfunction over following days. Figure 8B shows the concentration of APP in the brain ISF which increase 40, 370, and 800% for simulated exposures. Biomarker Aß_42_ follows the dynamics of APP with a noticeable delay, reaching a maximum around 1–1.4 days following the exposure (Figure 8C). The demarcation line at 2.5 above the physiological level has been to demonstrate the potential “window” for biomarker collection, with approximately 2 days post-mild blast exposure and 5 days for moderate exposure. The model will have to be calibrated, but the reported human data show similar time windows for plasma Aβ (Boutté et al., 2021). Lastly, our model predicts that a single mild insult cannot induce enough damage for a detectable brain injury.

## 4. Discussion

Billions of neurons in the human brain connected to one another by trillions of synapses are continuously subjected to mechanical forces communicated *via* adhesion molecules, cell membranes, extracellular and intracellular matrix. This endogenous physiological neuro-mechanobiology affects CNS cellular neurotransmission, metabolism and plasticity. However, external mechanical forces may cause synaptic structural damage and initiate a cascade of mechanical and biochemical deleterious and recovery responses. Mathematical models of synaptic mechanobiology supported by laboratory *in vitro* and *in vivo* experiments may help in better understanding of brain injury, diagnostics and protection. Mathematical modeling of CNS synaptic mechanobiology is challenging due to the immense number of brain synapses and heterogeneity of their morphologies as well as extreme range of spatial and temporal scales involved in brain injury responses (Procès et al., 2022). Structural mechanics of CAMs can be simulated at various scales using molecular and coarse-grained dynamics models, spring-mass-damper models or finite element type models. However, the spatial and time-step limitations make them impractical for longer duration (seconds to hours) simulation periods.

In this work, we formulated a dynamic model of a single synaptic structure using a network of ligand-receptor binding kinetics of synaptic CAMs and quasi-equilibrium energy-displacement model. The model can resolve time scales ranging from milliseconds during the hyperacute phase of mechanical loading to minutes-hours acute/chronic phase of injury progression/repair. Such synaptic mechanobiology model can be coupled with other models such as biomarker kinetics (BxK), neurotransmission, neuroimmune responses, and synaptic plasticity. Here we demonstrated a link between the synaptic mechanobiology model and a minimal model of synaptic processing of amyloid precursor protein and generation and kinetics of related amyloid biomarkers that can be detected in CSF and plasma.

First, we established our model’s capability in simulating the synaptic response to an acute injury characterized by a single separation of synapse over a 0.5 ms, almost equivalent to the positive phase duration of low-level blast waves. As a response to acute injury, our model predicted a viscoelastic-type behavior in which the synaptic cleft and force slowly restore their baseline values 8 min after the insult (Figure 3). During the synaptic cleft separation, initially a sharp spike is seen in the total forces generated in CAMs [marked by (*) in Figure 3B]. The sudden rise in force is due to the stretch of bound CAMs and their associated stiffnesses which resist the cleft separation. Conversely, the large generated force sharply increases the CAM complex unbinding rates which leads to their rapid dissociation, reduction in their concentrations, and eventually a decrease in force. The spike is then followed by a less steep reduction in force [marked by (^**^) in Figure 3B] which is the result of competition between unbinding due to remaining intermolecular force and distance-dependent rebinding of detached CAMs. The CAM forces vanish when the synapse cleft recovers its initial dimensions. However, since a significant loss is observed in SynC12 concentrations, a full recovery of synapse is not yet achieved (Figure 4B). In this work, SynC12 have a relatively higher dissociation constant and detachment parameter (*k*_*break*_), and thus, a greater portion of bound SynC12 are irreversibly detached from the membranes. The recovery of SynC12 requires natural turnover of unbound SynCAM1/2. Conversely, we assumed that NCAD do not detach from their cytoplasmic ends. Therefore, there is enough unbound NCAD on both sides of synapse to recover their baseline values within minutes after the acute insult.

To understand the effect of model parameters on the simulation results, a global sensitivity analysis was performed (Figure 6). The analysis results indicated that *M* (a constant modulating the synapse response to free energy changes) and σ (width distribution of binding constants) are the most sensitive parameters. We must note that *M* is a parameter for the synapse structure, and σ is shared between different CAM families. Among CAM specific parameters, SynCAM parameters are generally more sensitive and NCAD parameters are less sensitive. This is expected since the model output is considered long-term synaptic dysfunction, while NCAD complexes recover their initial concentrations minutes after the insult.

Next, we extended our model to simulate the synaptic response to multiple LLBs within a timeframe comparable to military weapon training scenarios. Exposure to repeated LLBs noticeably increases the long-term effects of damage. This is particularly evident in Figure 7 where after each insult, the XL and SynC12 concentrations progressively fall. Decreasing the frequency of exposures from every 10 min to every 30 min, however, did not have a significant effect on the long-term effects. Therefore, our model predicts that in a weapon training setting where shots are fired minutes apart, the number of exposures rather than their firing interval could be the determining factor in long-term effects of synaptic injury. We must note that while cumulative effects of exposure to repeated blasts on brain tissue integrity have been established (Trotter et al., 2015), the relationship between blast exposure frequency and brain pathology is currently unclear (Heyburn et al., 2021). Long-term recovery of CAMs and their complexes are largely modulated by the natural turnover of SynCAMs, NX, and NL. Cohen et al. (Cohen et al., 2013) reported half-lives of 2.56 and 2.63 days for SYNCAM1/2 and NX/NL, respectively. Accordingly, the loss of these CAMs is eventually recovered *via* their natural turn-over process after ∼3 months and ∼9 months for XL/NL and SynCAMs, respectively. We must note that this recovery time is predicted in absence of intrinsic homeostatic compensatory mechanisms which might expedite the CAM recoveries.

Finally, we illustrated the utility of our model in quantifying biomarker release and kinetics after exposure to repeated blasts (Figure 8). In this work, we only accounted for the amylogenic pathway shown in Figure 2. However, the non-amylogenic pathway could be useful for diagnostic, prophylactic, or therapeutic investigations of APP-affecting enzymes such as ADAM10 (Appel et al., 2021). Our results highlighted that the dynamics of biomarker (Aß_42_) following LLBs are demonstrably slower than the synaptic responses. This is particularly depicted in brain interstitial APP and plasma Aß_42_ peaking at around 1 and 2 days after the exposure, respectively. Moreover, our model predicts that the plasma biomarkers remain elevated for months after the initial exposure. Our findings of increased Aß_42_ in plasma 3–5 days post-blast exposure compare relatively well to recently reported human plasma Aß_42_ and Aß_40_ levels 1-, 2- and 3-days post-weapon blast exposure in military sniper training (Thangavelu et al., 2020; Boutté et al., 2021). We are collecting related human blast dose biomarker response data for more quantitative model validation. Several experimental studies have shown that the elevated of soluble Aß_42_ in TBI might induce oligomerization and aggregation leading to accelerated neurocognitive disease progression (e.g., Alzheimer’s disease) (Roberts et al., 1994; Washington et al., 2014). Taken together, these results underscore the importance computational models that delineate neuro*-response* and their link to clinical biomarker markers in LLB exposure.

In this work we have only considered mechanobiology of a single synapse in response to single and repeated blast injury. In the human CNS there are several other cellular and molecular adhesion structures susceptible to external loads including the axons, astrocytes, blood-brain barrier as well as extracellular matrix and intracellular cytoskeletal networks. The present “minimal” model of synaptic mechanobiology can be improved by accounting not only for tension loads but also shear loads. The model should be parametrized to account for variable morphology and composition of CAMs in excitatory and inhibitory synapses. Moreover, the present model is largely focused on the mechanics and kinetics CAM-CAM interactions. The mechanics of CAM connections to their respective synaptic membranes, however, is less understood. For instance, neuroligins and neurexins both have relatively short intracellular domains that potentially bind to several PDZ-domain scaffolding proteins (Meyer et al., 2004). This lack of mechanistic understanding of the proteins inside the cell motivated us to use a phenomenological pulling rate dependent down-regulation term for the detachment of SynCAMs and NX and NL from their synaptic membrane. This term is particularly crucial in understanding the recovery phase and biomarker kinetics. Further improvements could couple the present synapse model with reduced order mechanical models of the connected pre-synaptic axon segment and post-synaptic dendritic spine models e.g., spring-damper-mass models accounting for the elastic and damping effects of the extracellular matrix. The present mechanobiology model could be also adapted for modeling CAM effects between the axon and myelin junctions next to nodes of Ranvier and the adhesion dynamics between intraconal microtubule network and the microtubule-associated Tau proteins.

The present model needs to be calibrated and validated on experimental data collected from *in vitro* imaging of neuro-axonal structures and generation of injury related biomarkers. At present, we have linked the synaptic mechanobiology model with simple model of amyloid beta (Aß_42_) responses. More advanced models of generations of soluble synaptic biomarkers exist that could be incorporated in the present framework and validated on experimental data. Furthermore, several experimental and clinical studies have highlighted the neuro-immune responses such as neuroinflammation and microglial priming in repeated TBI (Kokiko-Cochran and Godbout, 2018). Accordingly, minimal or extended models of cytokine-mediate microglial activation from resting to pro- and anti-inflammatory phenotypes could be integrated in the presented framework to investigate neuroinflammation (Donat et al., 2017; Amato and Arnold, 2021).

## 5. Conclusion

Synaptic injury mechanisms are largely unknown and have only recently begun to attract interest of neuroscientists partially because of experimental challenges at such small length and timescales. Whether the synaptic injury is a primary result of the mechanical loads or a secondary effect of axonal injury remains to be determined. Most likely both mechanisms occur at the same time at different locations in the brain and induce mutual secondary effects (Jamjoom et al., 2021). This paper presented a novel reduced order model of synaptic mechanobiology caused by an acute and repeated blast brain injury. The model integrates the adhesion dynamics of the synaptic CAMs with the deformation mechanics of the synaptic cleft. Depending on the blast load level it predicts the reversible CAM adhesion recovery after lower level and slower rate loads and irreversible disconnect of some CAMs caused by bigger and faster rate loads. The model has been used to simulate the synaptic injury responses caused by repeated blast loads. It demonstrated the relevance of the duration of recovery period between repeated loads on the synaptic injury responses. In this work, we have linked the synaptic mechanobiology model with a minimal model of biomarker (Aβ_42_) kinetics. The model can be extended to simulate not only tension but also shear loads on both excitatory and inhibitory synapses, account for ECM effects and link to a synaptic neurotransmission model. Most importantly, the model needs to be further calibrated and validated on *in vitro* experimental data.

## Data availability statement

The original contributions presented in this study are included in this article/Supplementary material, further inquiries can be directed to the corresponding authors.

## Author contributions

HG and AP formulated the model. RG formulated modeling requirements for repeated blast TBI, CFDRC team (HG, HTG, ZC, and AP in equal contribution) implemented the model and conducted simulations and reviewed the results and the manuscript. All authors contributed to the article and approved the submitted version.
