# Supplementary material for: Mathematical model of mechanobiology of acute and repeated synaptic injury and systemic biomarker kinetics
Source: Front Cell Neurosci. 2023 Feb 6;17:1007062. doi: 10.3389/fncel.2023.1007062 (PMC9939777; doi:10.3389/fncel.2023.1007062)
Supplement: Supplementary file 1 [file Data_Sheet_1.pdf]

Supplementary Materials A – Synaptic Damage Model ODEs

The list of ODEs and parameters as they are used in the model are presented here.

1 Model parameters

zc = 2.000000e-02 # Same as z_0

sig = 1.300000e-02 #σ

k_off = 1.500000e-02 #k_{off}^0 XL

v_break = 1.000000e+00 #v_{break}

M = 1.000000e-08 #M

z_xl = 2.000000e-02 # Same as z_0

lam_xl = 1.250000e+03 #λ_{CAMs}

z0 = 2.000000e-02 #z_0

KD = 5.000000e+01 #k_D^0 XL

Cnx0 = 1.400000e+02 #C_{NX}^0

Cnl0 = 1.400000e+02 #C_{NL}^0

Cxl0 = 3.920000e+02 #C_{XL}^0

s_rate = 1.000000e+01 #$s(t)$

duration = 5.000000e-04 #Pulling duration

f0 = 4.000000e+03 #f_0 XL

k_rup = 3.090000e-06 #k_{rup} NX,NL

k_break = 1.000000e+01 #k_{break}

k_off_NCAD = 4.500000e-01 #k_{off}^0 NCAD:NCAD

k_on_NCAD = 2.300000e-03 #k_{on}^0 NCAD:NCAD

C_NCAD0 = 2.000000e+02 #C_{NCAD}^0

C_NCADNCAD0 = 2.044444e+02 #$C_{NCADNCAD}^0$

f0_NCAD = 5.000000e+02 #f_0 NCAD

k_rup_NCAD = 3.500000e-06 #k_{rup} NCAD

k_off_Syn = 1.500000e-02 #k_{off}^0 SynC12

KD_Syn = 9.360000e-01 #k_D^0 SynC12

C_Syn0 = 2.000000e+01 #$C_{SynCAM1,2}^0$

C_Syn120 = 4.273504e+02 #C_{SynC12}^0

f0_Syn12 = 4.000000e+03 #f_0 SynC12

k_rup_Syn = 3.090000e-06 #k_{rup} SynCAM1,2

k_break_Syn = 1.500000e+02 #k_{break} SynC12

2 ODEs and explicit function

NL:NX parameters & Equations

f_xl = lam_xl*(Cxl)*(z - z_xl)

k_on_b = k_off/KD

k_off_f = k_off*exp(f_xl/f0)

k_on = k_on_b*exp(-(z-zc)*(z-zc)/(2*sig*sig))

P = exp(-z_velocity/v_break)

k_rup_nx = k_rup

k_syn_nx = k_rup_nx*Cnx0

d(Cnx)/dt = -k_on*Cnx*Cnl + k_off_f*Cxl - k_rup_nx*Cnx + k_syn_nx

k_rup_nl = k_rup

k_syn_nl = k_rup_nl*Cnl0

d(Cnl)/dt = -k_on*Cnx*Cnl + k_off_f*Cxl - k_rup_nl*Cnl + k_syn_nl

dt(Cxl)/dt = +k_on*Cnx*Cnl - k_off_f*Cxl - (1 - P)*k_break*Cxl

NCAD parameters & Equations

f_NCADNCAD = lam_xl*(C_NCADNCAD)*(z - z_xl)

k_on_NCAD_e = k_on_NCAD*exp(-(z-zc)*(z-zc)/(2*sig*sig))

k_off_NCAD_e= k_off_NCAD*exp(f_NCADNCAD/f0_NCAD)

k_syn_NCAD = k_rup_NCAD*C_NCAD0

d(C_NCAD)/dt = -k_on_NCAD_e*C_NCAD*C_NCAD + k_off_NCAD_e*C_NCADNCAD - k_rup_NCAD*C_NCAD + k_syn_NCAD

d(C_NCADNCAD)/dt=+k_on_NCAD_e*C_NCAD*C_NCAD - k_off_NCAD_e*C_NCADNCAD

SynCAM parameters & Equations

f_Syn12 = lam_xl*(C_Syn12)*(z - z_xl)

k_on_Syn = k_off_Syn/KD_Syn

k_on_Syn_e = k_on_Syn*exp(-(z-zc)*(z-zc)/(2*sig*sig))

k_off_Syn_e = k_off_Syn*exp(f_Syn12/f0_Syn12)

k_syn_Syn = k_rup_Syn*C_Syn0

d(C_Syn)/dt = -k_on_Syn_e*C_Syn*C_Syn + k_off_Syn_e*C_Syn12 - k_rup_Syn*C_Syn + k_syn_Syn

d(C_Syn12)/dt= +k_on_Syn_e*C_Syn*C_Syn - k_off_Syn_e*C_Syn12 - (1 - P)*k_break_Syn*C_Syn12

Synapse deformation & dynamics

dFdz = lam_xl*(Cxl + C_NCADNCAD + C_Syn12)*(z-z_xl)

d(z)/dt = -M*(dFdz) + z_velocity

Supplementary Materials B – Biomarker kinetics model parameters and equations

The biomarker kinetics model is constructed from a series of reactions.

$$A \leftrightarrow B, \quad Forward\ reaction\ rate, \quad Reverse\ reaction\ rate$$

3 Brain ISF reactions

$$\emptyset \leftrightarrow APP, \quad k_{on}, \quad k_{off}$$

$$\emptyset \leftrightarrow \beta S, \quad k_{synth,\beta S}, \quad k_{clear,\beta S}$$

$$\emptyset \leftrightarrow \gamma S, \quad k_{synth,\gamma S}, \quad k_{clear,\gamma S}$$

$$\beta S \longrightarrow s\beta S, \quad k_{cleave,\beta S}$$

$$APP + \beta S \leftrightarrow APP{:}\beta S, \quad k_{onAPP}, \quad k_{off\beta S}$$

$$APP{:}\beta S \longrightarrow C99 + \beta S, \quad k_{cat,\beta S}$$

$$C99 + \gamma S \leftrightarrow C99{:}\gamma S, \quad k_{on,C99}, \quad k_{off,\gamma S}$$

$$C99{:}\gamma S \longrightarrow A\beta_{bisf} + \gamma S, \quad k_{cat,\gamma S}$$

$$A\beta_{bisf} \longrightarrow \emptyset, \quad k_{clear,bisf}$$

4 Monomer transport and degradation in the body

$$A\beta_{plasma} \leftrightarrow A\beta_{bisf}, \quad k_{14}, \quad k_{41}$$

$$A\beta_{bisf} \longrightarrow A\beta_{csf}, \quad k_{43}$$

$$A\beta_{plasma} \leftrightarrow A\beta_{csf}, \quad k_{13}, \quad k_{31}$$

$$A\beta_{plasma} \leftrightarrow A\beta_{peripheral}, \quad k_{12}, \quad k_{21}$$

$$A\beta_{plasma} \longrightarrow \emptyset, \quad k_{clear,plasma}$$

$$A\beta_{peripheral} \longrightarrow \emptyset, \quad k_{clear,peripheral}$$

5 Nomenclature

APP: amyloid precursor protein

βS: β-secretase

$s\beta S$: soluble β-secretase

$C99$: 99 amino acid C-terminal fragment of Amyloid Precursor Protein APP

γS: γ-secretase

$A\beta_{bisf}$: amyloid beta monomers in brain interstitial fluid

$A\beta_{csf}$: amyloid beta monomers in cerebrospinal fluid

$A\beta_{peripheral}$: amyloid beta monomers in peripheral tissue

$A\beta_{plasma}$: amyloid beta monomers in plasma

The list of ODEs and parameters as they are used in the model are presented here.

6 Model parameters

s = 1 #time unit

L = 1 #volume unit

kon_Bx_0 = 0.1 #$K^{0}_{BISF,synth}$

kon_Bx_S = 1e1 #K_{SD}

koff_Bx = 0.0001 #k_{off}

SD = 1*abs(Cxg-Cxg0)/(Cxg0) + 0.1*abs(C_Syn12-C_Syn120)/(C_Syn120) #Synaptic dysfunction

kon_Bx = kon_Bx_0+kon_Bx_S*SD #k_{on}

k_onPP = 1.0E-3 #k_{onAPP},

k_Vplasma = 3 #Plasma volume

k_Vperipheral = 3 #Peripheral volume

k_Vcsf = 0.139 #CSF volume

k_Vbisf = 0.261 #Brain ISF volume

k_synthBACE_bisf = 1.0E-4/k_Vbisf #$k_{synth,\beta S}$

k_clearBACE = 1.2E-5 #$k_{clear,\beta S}$

k_synthGamma_bisf = 5.2E-5/k_Vbisf #$k_{synth,\gamma S}$

k_clearGamma = 8.0E-6 #$k_{clear,\gamma S}$

k_cleave = 2E-7 #$k_{cleave,\beta S}$

k_offBACE = 120 #$k_{off\beta S}$

k_catBACE = 7.2E-3 #$k_{cat,\beta S}$

k_offGamma = 0.4 #$k_{off,\gamma S}$

k_catGamma = 1.2E-3 #$k_{cat,\gamma S}$

k_clearBx_bisf = 5.5e-5 #$k_{clear,bisf}$

k_clearBx_plasma = 1.9E-4 #$k_{clear,plasma}$

k_12Bx = 3E-4 # k_{12}

k_21Bx = 2.0E-5 #k_{21}

k_13Bx = 1.72E-9 #k_{13}

k_31Bx = 4.5E-5 #k_{31}

k_14Bx = 1.48E-7 #k_{14}

k_41Bx = 1.48E-8 #k_{41}

k_43Bx = 7.5E-5 #k_{43}

7 Rate equations

In all the equations below, Bx, BACE, and CTFbeta, are $Aß_{42}$ monomers, ß-secretase, and C99, respectively.

d(APP_bisf)/dt = (kon_Bx-koff_Bx*APP_bisf) - (k_onPP*APP_bisf*BACE_bisf-k_offBACE*APP_BACE_bisf)

d(BACE_bisf)/dt = (k_synthBACE_bisf-k_clearBACE*BACE_bisf) - k_cleave*BACE_bisf - (k_onPP*APP_bisf*BACE_bisf-k_offBACE*APP_BACE_bisf) + k_catBACE*APP_BACE_bisf

d(gamma_bisf)/dt = (k_synthGamma_bisf-k_clearGamma*gamma_bisf) - (k_onPP*gamma_bisf*CTFbeta_bisf-k_offGamma*CTFbeta_gamma_bisf) + k_catGamma*CTFbeta_gamma_bisf

d(BACEs_bisf)/dt = k_cleave*BACE_bisf

d(APP_BACE_bisf)/dt = (k_onPP*APP_bisf*BACE_bisf-k_offBACE*APP_BACE_bisf) - k_catBACE*APP_BACE_bisf

d(CTFbeta_bisf)/dt = k_catBACE*APP_BACE_bisf - (k_onPP*gamma_bisf*CTFbeta_bisf-k_offGamma*CTFbeta_gamma_bisf)

d(CTFbeta_gamma_bisf)/dt = (k_onPP*gamma_bisf*CTFbeta_bisf-k_offGamma*CTFbeta_gamma_bisf) - k_catGamma*CTFbeta_gamma_bisf

d(Bx_bisf)/dt = k_catGamma*CTFbeta_gamma_bisf - k_clearBx_bisf*Bx_bisf + (k_14Bx*Bx_plasma-k_41Bx*Bx_bisf) - k_43Bx*Bx_bisf

d(Bx_plasma)/dt = -k_clearBx_plasma*Bx_plasma - (k_12Bx*Bx_plasma-k_21Bx*Bx_peripheral) - (k_13Bx*Bx_plasma-k_31Bx*Bx_csf) - (k_14Bx*Bx_plasma-k_41Bx*Bx_bisf)

d(Bx_peripheral)/dt = -k_clearBx_plasma*Bx_peripheral + (k_12Bx*Bx_plasma-k_21Bx*Bx_peripheral)

d(Bx_csf)/dt = (k_13Bx*Bx_plasma-k_31Bx*Bx_csf) + k_43Bx*Bx_bisf

Supplementary Materials C – Long-term recovery of CAMs

In this section, we ran the repeated blast exposure simulation Case 1 (every 10 minutes) for almost 40 months after the initial insult. Long-term recovery of the CAMs is shown in **Figure B1**. NCAD:NCAD immediately returns back to original concentration. NX:NL complexes take a few months to recover. SynCAM12 takes months to return to original levels. The main parameter controlling the long-term response of SynCAM12 is their synthesis and rupture rates. Alternative homeostatic mechanisms, not included here, might play a role in expediting the recovery of lost CAMs.

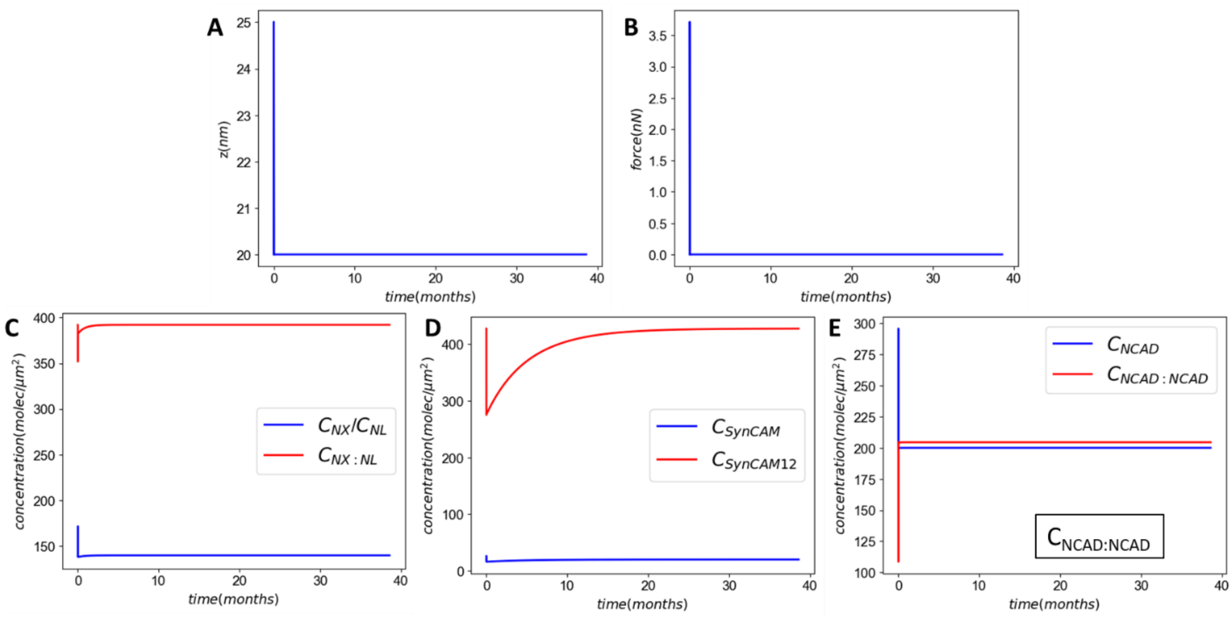

Figure B1. Panel A shows the synaptic cleft size. Panel B shows the total force. Panels C, D, and E show the concentration of CAMs (bound and unbound) for ~40 months simulation.
